# Heat Waves and Adverse Health Events Among Dually Eligible Individuals 65 Years and Older

**DOI:** 10.1001/jamahealthforum.2024.3884

**Published:** 2024-11-08

**Authors:** Hyunjee Kim, Eun-Hye Yoo, Angela Senders, Clint Sergi, Hiroko H. Dodge, Sue Anne Bell, Kyle D. Hart

**Affiliations:** 1 Center for Health Systems Effectiveness, Oregon Health & Science University, Portland; 2 Department of Geography, University at Buffalo, Buffalo, New York; 3 Department of Neurology, Massachusetts General Hospital, Harvard Medical School, Boston; 4 University of Michigan School of Nursing, Ann Arbor

## Abstract

**Importance:**

Extensive research has found the detrimental health effects of heat waves. However, a critical gap exists in understanding their association with adverse health events among older dually eligible individuals, who may be particularly susceptible to heat waves.

**Objective:**

To assess the association between heat waves and adverse health events among dually eligible individuals 65 years and older.

**Design, Setting, and Participants:**

This retrospective time-series study assessed the association between heat waves in warm months from 2016 to 2019 and zip code tabulation area (ZCTA)–day level adverse health events. Dually eligible individuals 65 years and older who were continuously enrolled in either a Medicare fee-for-service plan or a Medicare Advantage plan with full Medicaid benefits from May to September in any given year were identified. All ZCTAs in the US with at least 1 dually eligible individual in each study year were included. Data were analyzed from September 2023 to August 2024.

**Exposure:**

Heat waves, defined as 3 or more consecutive extreme heat days (ie, days with a maximum temperature of at least 90 °F [32.2 °C] and in the 97th percentile of daily maximum temperatures for each ZCTA during the study period).

**Main Outcomes and Measures:**

Daily counts of heat-related emergency department visits and heat-related hospitalizations for each ZCTA.

**Results:**

The study sample included 5 448 499 beneficiaries 65 years and older in 28 404 ZCTAs across 50 states and Washington, DC; the mean (SD) proportion of female beneficiaries and beneficiaries 85 years and older in each ZCTA was 66% (7%) and 20% (8%), respectively. The incidence rate for heat-related emergency department visits was 10% higher during heat wave days compared to non–heat wave days (incidence rate ratio [IRR], 1.10; 95% CI, 1.08-1.12), and the incidence rate of heat-related hospitalizations was 7% higher during heat wave days (IRR, 1.07; 95% CI, 1.04-1.09). There were similar patterns in other adverse health events, including a 4% higher incidence rate of death during heat wave days (IRR, 1.04; 95% CI, 1.01-1.07). The magnitude of these associations varied across some subgroups. For example, the association between heat waves and heat-related emergency department visits was statistically significant only for individuals in 3 of 9 US climate regions: the Northwest, Ohio Valley, and the West.

**Conclusions and Relevance:**

In this time-series study, heat waves were associated with increased adverse health events among dually eligible individuals 65 years and older. Without adaptation strategies to address the health-related impacts of heat, dually eligible individuals are increasingly likely to face adverse outcomes.

## Key Points

**Question:**

Is there an association between heat waves and adverse health events among dually eligible individuals 65 years and older?

**Findings:**

In this time-series study of 5.5 million dually eligible individuals, exposure to heat waves was associated with increased heat-related emergency department visits, heat-related hospitalizations, and deaths.

**Meaning:**

Without adaptation strategies, older dually eligible individuals 65 years and older are increasingly likely to face adverse health events as extreme heat events occur more frequently.

## Teaser

This time-series study assesses the association between heat waves and adverse health events among dually eligible individuals 65 years and older.

## Introduction

Heat waves, defined as prolonged periods of unusually hot weather, pose an urgent threat to human health.^1,2^ While individual heat wave events, such as the record-setting temperatures experienced in the Pacific Northwest in 2021^3^ and the Southwest in 2023,^4^ draw public attention for their toll on human life, their impact extends beyond fatalities. Heat waves are associated with a wide range of adverse health events, including increased hospitalizations and emergency department (ED) visits, as well as the exacerbation of existing chronic conditions, particularly among older adults.^5,6,7,8^

While extensive research has found detrimental effects of heat waves, a gap exists in the understanding of their association with adverse health outcomes among individuals 65 years and older who are dually eligible for Medicare and Medicaid benefits. This population, representing about 8 million individuals in the US,^9,10^ is likely to be highly susceptible to heat waves: 90% have an annual income below $20 000; 50% report fair or poor health, live with mental health challenges, or experience limitations in activities of daily living^9^; and 40% live alone.^11^ While each of these factors alone can exacerbate the challenges posed by heat waves,^12^ dually eligible individuals often face multiple risk factors simultaneously.

However, the situation is complex. A substantial proportion of dually eligible individuals (13% vs 1% for older adults without dual eligibility^9^) reside in nursing facilities. These facilities are required by federal regulations to maintain interior temperatures between 71 and 81 °F,^13^ which may help reduce heat wave–related adverse health outcomes. Investigating this complex interplay of risks and protective factors, Visaria et al,^14^ in their examination of the extreme heat impact on all Medicare beneficiaries, found an association between extreme heat and adverse health events among dually eligible individuals. This finding underscores the importance of further research to understand the specific vulnerabilities of this population during heat waves.

We assessed the association between heat waves and adverse health events among dually eligible individuals 65 years and older. We hypothesized that heat waves would be associated with a higher likelihood of adverse health events and that this association would vary across subgroups by demographic, socioeconomic, and health-related factors. Understanding the association of heat waves among dually eligible individuals and across subgroups can inform more targeted adaptation efforts.^15^

## Methods

### Data

We created a database comprising daily weather data^16^ and daily counts of adverse health events for each zip code tabulation area (ZCTA), with ZCTA-day as the unit of analysis. We selected the ZCTA as the spatial unit of analysis due to variability in heat wave occurrences within larger geographic areas, such as counties, which have been frequently used as the unit of analysis in previous studies.^5,17,18,19,20^ For example, ZCTAs within California counties exhibited different cumulative numbers of heat wave days during the study period (eFigure 1 in Supplement 1). The ZCTA was the most granular geographic level at which we could count adverse health events in the data.

The data included claims and weather data from May to September (because heat waves are most prevalent during these months) in 2016 to 2019. We analyzed the national Medicare Master Beneficiary Summary File, Medicare fee-for-service claims, Medicare Advantage encounters, and Medicaid claims, linked at the person level. We used the Medicare Master Beneficiary Summary File to identify dually eligible individuals at least 65 years old. We used Medicare fee-for-service or Medicare Advantage encounter files to identify hospitalizations and ED visits for each dually eligible individual and used Medicaid claims to identify long-term nursing facility placements. We obtained daily temperature and relative humidity data from Daymet (Oak Ridge National Laboratory Distributed Active Archive Center),^16^ which provides data points at a resolution of 1 km × 1 km (eTable 1 in Supplement 1; a guide to the R codes used to obtain weather data is included in eAppendix 1 in Supplement 1).

The Oregon Health & Science University institutional review board approved this study with a waiver of informed consent, as seeking informed consent from all patients included in the study was not feasible and the risk to study participants was minimal. We followed the Strengthening the Reporting of Observational Studies in Epidemiology (STROBE) reporting guidelines.

### Study Sample

We first identified 6 279 148 dually eligible individuals 65 years and older who were continuously enrolled in either a Medicare fee-for-service plan or a Medicare Advantage plan with full Medicaid benefits from May to September in any given year, provided that they were alive during that period. Subsequently, we excluded individuals who lived in US territories, relocated across counties within a year, or had inconsistent ZCTAs or dual-eligibility status in Medicare and Medicaid records. We included all ZCTAs with at least 1 dually eligible individual in each study year. This yielded 5 448 499 unique dually eligible individuals. We aggregated this data to the ZCTA-day level, resulting in a final sample of 28 404 ZCTAs in 50 states and Washington, DC (eTable 2 in Supplement 1).

### Outcomes

Primary outcomes included ZCTA-level daily counts of heat-related ED visits and heat-related hospitalizations. We classified an ED visit or hospitalization as heat related if any claim or encounter contained *International Statistical Classification of Diseases and Related Health Problems, Tenth Revision,* diagnosis codes relevant to conditions influenced by heat exposure (eAppendix 2 in Supplement 1).^21,22^

Secondary outcomes included ZCTA-day counts of all-cause ED visits, heat-specific ED visits, all-cause hospitalizations, heat-specific hospitalizations, deaths, and long-term nursing facility placements within 3 months following a heat wave. We classified ED visits and hospitalizations as heat specific if any claims contained diagnosis codes that indicated direct heat or light exposure, a narrower definition than heat-related diagnoses. We defined nursing facility placements as a Medicaid-covered nursing facility claim with no prior stay in the previous year. We considered nursing facility placement as an outcome because individuals exposed to heat waves may experience accelerated cognitive or functional decline, potentially leading to their entrance into a nursing facility.

### Heat Wave Day

We converted 1 km × 1 km daily maximum temperature data to ZCTA-level daily maximum temperature by averaging the values from all grid cells within or intersecting with a ZCTA boundary. We then used a combination of absolute and relative thresholds to identify extreme heat days from May to September in 2016 to 2019.^20,21,23^ Specifically, we defined a single day of extreme heat for a ZCTA as a day with a maximum temperature of at least 90 °F (32.2 °C) and in the 97th percentile of daily maximum temperatures for that ZCTA during the study period. We defined a heat wave as 3 or more consecutive extreme heat days.

To capture the impacts of the intensity and duration of heat waves, we considered additional heat wave definitions. We redefined a single day of extreme heat for a ZCTA as having a maximum daily temperature of at least 90 °F and being in the 90th percentile of maximum daily temperatures for that ZCTA during the study period. Additionally, we varied the duration of heat waves to include at least 1, 2, 3, or 4 consecutive extreme heat days for both definitions of an extreme heat day.

It is possible that extreme heat exposure on one day may lead to adverse health events in the following days.^5^ To address this, we assessed whether the association between heat wave exposure and heat-related ED visits and hospitalizations persisted beyond the end of a heat wave by extending heat wave indicators to 1 to 5 days beyond the end of each heat wave.

### Statistical Analysis

We fit separate multivariable Poisson regression models for each outcome to assess the association between heat waves and adverse health events. We chose Poisson regression because the outcomes were counts without evidence of overdispersion. The results did not differ meaningfully in a sensitivity analysis using negative binomial models.

The measure of association was an incidence rate ratio (IRR) of the heat wave indicator. The model included fixed effects for ZCTA to account for time-invariant confounders across ZCTAs. Additionally, we included binary indicators for day of the week and federal holidays to account for their potential influence on the use of acute health services such as ED visits. Furthermore, we included indicators of each week of the study period (ie, week-year) to adjust for secular time trends common across all ZCTAs. Each model was additionally adjusted for ZCTA-level daily average relative humidity (from Daymet^16^), ZCTA-level annual proportion of female beneficiaries, and ZCTA-level annual proportion of individuals aged 65 to 74, 75 to 84, or 85 years and older. We clustered standard errors at the ZCTA, and all models were offset by the ZCTA annual population to account for differences in the size of the population at risk (eAppendix 3 in Supplement 1).

To identify dual-eligible individuals who may be at increased risk of adverse health events from heat waves, we conducted additional analyses stratified by Alzheimer disease and related dementias diagnosis among community-dwelling individuals (yes/no), substance use disorder diagnosis (yes/no), mental health diagnosis (none, mild to moderate, or severe mental illness), ZCTA rurality (urban/rural), ZCTA social deprivation index (lower/higher, with higher indicating more socially deprived areas),^24^ long-term care use (none, home- and community-based service use, or long-term nursing facility resident),^25,26,27^ race and ethnicity (Asian or Pacific Islander, Black, Hispanic, and White, as reported in Medicare data), and 9 climate regions identified by National Centers for Environmental Information (Northeast, Northern Rockies, Northwest, Ohio Valley, South, Southeast, Southwest, Upper Midwest, and West; eFigure 2 in Supplement 1).^28^ These subgroups are pertinent within the study population due to the high prevalence of conditions like dementias, along with unique health service use patterns (eg, long-term care use), which may influence individuals’ susceptibility to heat waves. Additionally, examining subgroups based on factors like social deprivation, race and ethnicity, and region could provide insight into variations in susceptibility to heat waves (eAppendices 4 and 5 and eTables 3 and 4 in Supplement 1).

We conducted a supplementary analysis to address the known issue of incomplete Medicare Advantage encounter data. We excluded beneficiaries enrolled in Medicare Advantage contracts with high rates of missing data^29^ and repeated the main analysis.

We used R, version 4.3.2 (R Project for Statistical Computing), for analyses and a 2-sided *P* < .05 as a threshold for statistical significance. Data were analyzed from September 2023 to August 2024.

## Results

This analysis included 5 448 499 beneficiaries 65 years and older living in 28 404 ZCTAs across 50 states and Washington, DC. The mean (SD) proportion of female beneficiaries residing in each ZCTA was 66% (7%), and the mean (SD) proportion of beneficiaries 85 years or older was 20% (8%) (eTable 5 in Supplement 1).

Exposure to heat waves (defined as 3 or more consecutive days with temperature above the 97th percentile) was associated with an increase in adverse health events (Table). Specifically, the incidence rates for heat-related ED visits and hospitalizations were 10% and 7% higher, respectively, during heat wave days compared to non–heat wave days (ED visits: IRR, 1.10; 95% CI, 1.08-1.12; hospitalizations: IRR 1.07; 95% CI, 1.04-1.09). There were similar patterns among other adverse health events, including a 4% higher incidence rate of death during heat wave days (IRR, 1.04; 95% CI, 1.01-1.07). However, there was no statistically significant difference in the incidence rates for long-term nursing facility placement between heat wave and non–heat wave days (IRR, 1.00; 95% CI, 0.98-1.02).

**Table.** Association Between Heat Waves and Adverse Health Events Among Dually Eligible Beneficiaries, 2016-2019^a^

Outcome	No. of adverse health events per 1 million beneficiary-days		Adjusted association between heat waves and adverse events, IRR (95% CI)
	Non–heat wave days	Heat wave days	
Primary outcomes			
Heat-related ED visits	537	584	1.10 (1.08-1.12)
Heat-related hospitalizations	451	467	1.07 (1.04-1.09)
Secondary outcomes			
All-cause ED visits	2718	2681	1.02 (1.01-1.03)
All-cause hospitalizations	1071	1058	1.04 (1.02-1.05)
Heat-specific ED visits	2	21	4.98 (4.42-5.60)
Heat-specific hospitalizations	1	7	6.36 (5.19-7.79)
NF placements within 3 mo	3402	2441	1.00 (0.98-1.02)
Deaths	237	239	1.04 (1.01-1.07)

Abbreviations: ED, emergency department; IRR, incidence rate ratio; NF, nursing facility.

^a^ A heat wave was defined as 3 or more consecutive extreme heat days with a maximum temperature of 90 °F (32.2 °C) or higher and in the 97th percentile of daily maximum temperatures for that zip code tabulation area during the study period.

**Figure 1.**
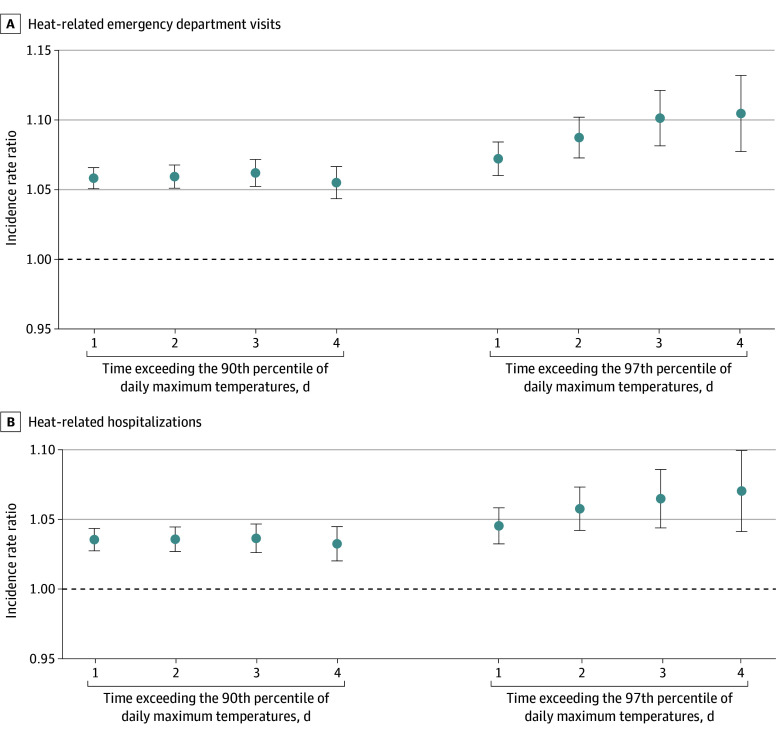
Association Between Heat Waves and Adverse Health Events Among Dually Eligible Beneficiaries by the Intensity and Duration of Heat Waves, 2016-2019 Heat waves were defined by 2 intensity thresholds: the 90th and 97th percentiles of daily maximum temperatures for each zip code tabulation area (ZCTA) during the study period. Additionally, duration of heat waves was varied to include at least 1, 2, 3, or 4 consecutive extreme heat days. Each point on the graph represents a different combination of intensity and duration. The incidence rate ratio estimate indicates the ratio of incidence rates for each outcome between heat wave days (all days during the heat wave period, from day 1 to the last day) and non–heat wave days (all days outside of the heat wave period). The Poisson regression models were adjusted for binary variables of ZCTA, day of the week, federal holidays, week of the study period, daily ZCTA-level relative humidity, and annual ZCTA-level demographics (proportion of female beneficiaries; age groups of 65-74, 75-84, and ≥85 years). Standard errors were clustered at the ZCTA level, and models were offset by annual ZCTA-level population. An incidence rate ratio higher than 1 (dashed line) indicates a higher incidence rate of the outcome during heat wave days compared to non–heat wave days. Error bars represent 95% CIs.

Regardless of duration and intensity, heat waves were consistently associated with increases in adverse health events, especially during at least 3- and 4-day–long heat waves when using the 97th percentile of daily maximum temperatures (Figure 1). For primary outcomes, the IRR was highest during the heat wave alone (0-day lag), except for heat-related hospitalizations (1-day lag, including 1 day after the end of the heat wave) (eFigure 3 in Supplement 1).

**Figure 2.**
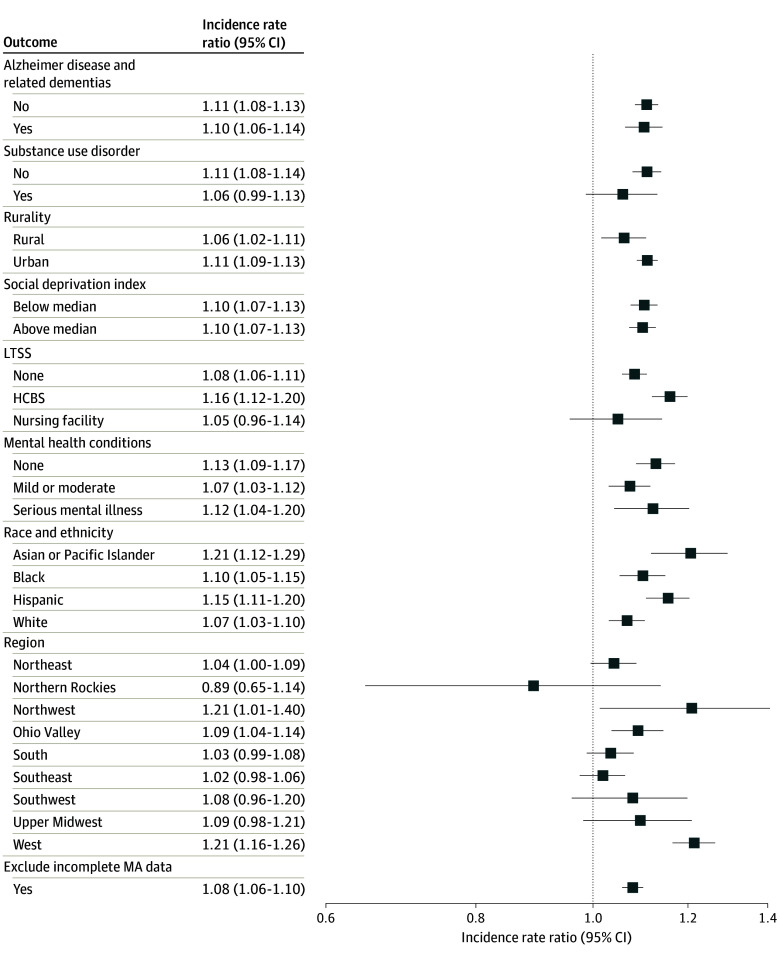
Subgroup Analyses of Heat Waves and Heat-Related Emergency Department Visits Among Dually Eligible Beneficiaries, 2016-2019 A single day of extreme heat was defined as a day with a maximum temperature of 90 °F (32.2 °C) or higher and in the 97th percentile of daily maximum temperatures for that zip code tabulation area (ZCTA) during the study period. A heat wave was defined as 3 or more consecutive extreme heat days. Incidence rate ratio estimates indicate the ratio of incidence rates for heat-related emergency department visits between heat wave and non–heat wave days for each subgroup. An incidence rate ratio higher than 1 indicates a higher incidence rate during heat wave days compared to non–heat wave days. The Poisson regression models adjusted for binary variables of ZCTA, day of the week, federal holidays, week of the study period, daily ZCTA-level relative humidity, and annual ZCTA-level demographics (proportion of female beneficiaries; age groups of 65-74, 75-84, and ≥85 years). Standard errors were clustered at the ZCTA level and included an offset for annual ZCTA-level population. Alzheimer disease and related dementias (yes/no) analyses excluded nursing home residents due to the high proportion of this population residing in facilities with mandated temperature control. Long-term services and supports (LTSS) analyses excluded 5 states (Arkansas, Idaho, Mississippi, New Hampshire, and Rhode Island) with unreliable long-term care claim volume, as indicated by the DQ Atlas.^26^ Regions were defined according to the National Centers for Environmental Information classification. HCBS indicates home- and community-based services; MA, Medicare Advantage.

**Figure 3.**
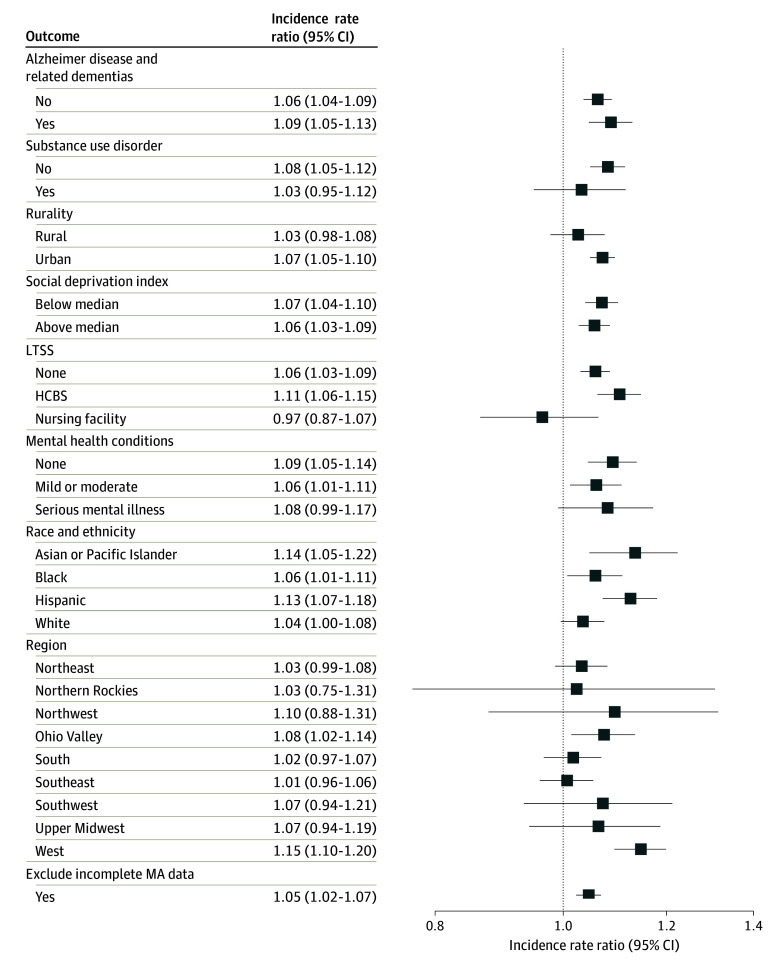
Subgroup Analyses of Heat Waves and Heat-Related Hospitalizations Among Dually Eligible Beneficiaries, 2016-2019 A single day of extreme heat was defined as a day with a maximum temperature of 90 °F (32.2 °C) or higher and in the 97th percentile of daily maximum temperatures for that zip code tabulation area (ZCTA) during the study period. A heat wave was defined as 3 or more consecutive extreme heat days. Incidence rate ratio estimates indicate the ratio of incidence rates for heat-related hospitalizations between heat wave and non–heat wave days for each subgroup. An incidence rate ratio higher than 1 indicates a higher incidence rate during heat wave days compared to non–heat wave days. The Poisson regression models adjusted for binary variables of ZCTA, day of the week, federal holidays, week of the study period, daily ZCTA-level relative humidity, and annual ZCTA-level demographics (proportion of female beneficiaries; age groups of 65-74, 75-84, and ≥85 years). Standard errors were clustered at the ZCTA level and included an offset for annual ZCTA-level population. Alzheimer disease and related dementias (yes/no) analyses excluded nursing home residents due to the high proportion of this population residing in facilities with mandated temperature control. Long-term services and supports (LTSS) analyses excluded 5 states (Arkansas, Idaho, Mississippi, New Hampshire, and Rhode Island) with unreliable long-term care claim volume, as indicated by the DQ Atlas.^26^ Regions were defined according to the National Centers for Environmental Information classification. HCBS indicates home- and community-based services; MA, Medicare Advantage.

In subgroup analyses, the incidence of heat-related ED visits was higher during heat wave days compared to non–heat wave days across most subgroup strata (Figure 2). For instance, all racial and ethnic groups exhibited higher incidence rates of heat-related ED visits during heat waves, with Asian beneficiaries experiencing a particularly elevated IRR of 1.21 (95% CI, 1.12-1.29). However, exceptions were noted, including no statistically significant difference in the incidence rate of heat-related ED visits between heat wave and non–heat wave days for long-term nursing facility residents (IRR, 1.05; 95% CI, 0.96-1.14). Additionally, incidence rates were statistically significantly higher on heat wave days among ZCTAs in 3 of 9 regions: the Northwest (Idaho, Oregon, and Washington; IRR, 1.21; 95% CI, 1.01-1.40), Ohio Valley (Illinois, Indiana, Kentucky, Missouri, Ohio, Tennessee, and West Virginia; IRR, 1.09; 95% CI, 1.04-1.14), and the West (California and Nevada; IRR, 1.21; 95% CI, 1.16-1.26). There was a similar pattern for heat-related hospitalizations (Figure 3). After excluding those enrolled in Medicare Advantage contracts with high rates of missing data, results remained largely unchanged for both heat-related ED visits and hospitalizations.

## Discussion

Analyzing nationwide Medicare and Medicaid claims combined with temperature data from 2016 to 2019, we found that heat waves were associated with increased adverse events among dually eligible individuals 65 years and older. The magnitude of this association varied among some subgroups. For example, the association between heat waves and heat-related ED visits was statistically significant only for individuals living in homes or communities, as opposed to nursing facilities, and for individuals residing in 3 of 9 climate regions: the Northwest, Ohio Valley, and the West.

This analysis builds on literature investigating adverse events associated with heat waves among older adults. For example, Bobb et al,^5^ using nationwide Medicare fee-for-service claims from 1999 to 2010, reported increased rates of hospitalizations for conditions including fluid and electrolyte disorders, heat stroke, septicemia, kidney failure, and urinary tract infection during heat waves. Similarly, Sun et al^6^ analyzed 2010 to 2019 commercial insurance claims and reported a higher risk of ED visits for any cause, heat-related illness, kidney disease, and mental disorders among older adults. Visaria et al^14^ focused on single extreme heat days (rather than heat waves) and analyzed 2008 to 2019 Medicare fee-for-service claims, finding increased heat-related ED visits on extreme heat days, especially among dually eligible individuals. The present study extends this body of existing studies by investigating the association between heat waves and a broad set of adverse outcomes, specifically among dually eligible individuals as well as various relevant subgroups.

A particularly interesting result was the statistically significant increase in mortality during heat waves. The incidence rate for death was 4% higher during heat wave days compared to non–heat wave days (IRR, 1.04; 95% CI, 1.01-1.07). This result is particularly noteworthy because increased mortality represents a tangible and severe health outcome that highlights the serious health risks that heat waves pose to dually eligible older adults. While this result is consistent with previous findings on heat-related mortality among older adults,^30,31^ it specifically emphasizes the increased susceptibility of dually eligible individuals during heat waves.

Consistent with our hypothesis, we found substantial increases in all other adverse events during heat waves, with the exception of nursing facility placements. While this finding may suggest that individuals exposed to heat waves were not necessarily more prone to entering a nursing facility due to accelerated cognitive or functional decline, it is important to interpret this result cautiously. Nursing facility entry within 3 months following a heat wave represents a long-term outcome, unlike short-term outcomes such as hospitalizations or ED visits, which occur during or relatively soon after heat waves. To address the long-term nature of nursing facility placement, we altered heat wave indicators in this analysis to extend up to 3 months beyond the end of each heat wave. However, this approach may not fully capture how nursing facility entry rates change over time as a result of heat waves. Therefore, further studies using alternative approaches would be beneficial for understanding the association between nursing facility placement and heat waves.

In the subgroup analyses, the incidence of heat-related ED visits and hospitalizations was generally higher during heat wave days compared to non–heat wave days across most subgroups, regardless of the presence of certain diagnoses such as Alzheimer disease and related dementias, substance use disorder, or mental health conditions, as well as different racial and ethnic backgrounds and varying levels of neighborhood social deprivation. This finding was unexpected because prior studies have identified these factors as contributors to heat-related adverse health events.^8,32,33,34^ One explanation for these findings is that dually eligible individuals often encounter a myriad of physical, mental, social, and financial challenges. Thus, focusing solely on one factor, such as dementia, may overlook the complex interplay of various risk factors.

However, there were exceptions in the subgroup analyses. For example, heat waves were not associated with increased incidence rates of heat-related ED visits or hospitalizations among nursing facility residents, whereas they were for those who received no long-term care or received care in home- or community-based settings. This finding offers reassurance, as nursing facility residents are likely to have chronic conditions such as heart disease, diabetes, or dementia; take medications like antipsychotics; or experience limited mobility,^35,36,37,38^ all of which can exacerbate the negative effects of heat waves. The lack of observed association between heat waves and adverse events for this group may be due to a federal regulation that mandates nursing facilities to maintain an interior temperature range of 71 to 81 °F.^13^ In contrast, incidence rates of adverse health outcomes were elevated for dually eligible individuals receiving care in home- or community-based settings during heat waves. Unlike nursing facility regulation, temperature control in community-based facilities varies and depends on state regulations.^39,40^

We observed another exception by climate region. While heat waves were associated with elevated adverse events in a few regions, notably the West (California and Nevada), the association was not as pronounced in other regions that tend to experience frequent heat waves. This could also explain the elevated IRRs for the Asian and Hispanic enrollees in the study sample, nearly half of whom lived in the West. Many Californians who live in coastal areas are accustomed to milder temperatures and lack air conditioning, making them susceptible during heat waves.^41^ In contrast, people who reside in traditionally hot regions may be more acclimated to extreme heat, with many more buildings equipped with air conditioning. Further research is warranted to understand why certain regions experienced a more pronounced association of adverse outcomes with heat waves, as this knowledge will inform targeted adaptation strategies.

### Limitations

This study has limitations. First, we relied on a claims database to identify adverse events, but there may be omissions in coding, particularly for heat-related conditions if clinicians have not widely adopted the use of *International Statistical Classification of Diseases and Related Health Problems, Tenth Revision,* codes for heat-related symptoms. Second, we did not adjust for variations in air quality or green space, which could confound the association of interest. Third, we could not consider variations in indoor heat exposures or adaptive behaviors (eg, air conditioning use). Fourth, while the fixed-effect regression models account for time-invariant confounding and secular time trends, the study results cannot be understood as causal. Fifth, the analysis could not compare the association between heat waves and adverse events among dually eligible individuals vs those without dual eligibility because the database contained claim data exclusively for dually eligible individuals. Finally, the subgroup analyses included 3 health conditions (dementia, substance misuse, and mental health), which do not fully capture the broader medical complexity of individuals in the sample.

## Conclusions

In this time-series study, heat waves were associated with increased adverse events among dually eligible individuals 65 years and older, with varying associations across subgroups. These findings underscore the vulnerability of this population during heat waves and emphasize the need for tailored adaptation strategies to prepare for the projected increase in intense and prolonged heat wave events.

## Notes

Supplement 1. **eFigure 1.** Local variation in heatwave days across ZCTAs within county boundaries in California **eTable 1.** Data sources utilized in this study **eAppendix 1.** Guide to R code **eTable 2.** Study sample selection **eAppendix 2.** Identification of outcomes **eAppendix 3.** Model specification **eFigure 2.** Nine climate regions by national centers of environmental information **eAppendix 4.** Identification of subgroups **eTable 3.** Type of service codes and place of service codes used to identify long-term service and supports (LTSS) use **eAppendix 5.** Subgroup sample selection **eTable 4.** Sample selection for subgroup analyses **eTable 5.** Zip code tabulation area (ZCTA)-level descriptive statistics, 2016-2019 **eFigure 3.** Association between lagged heatwaves and adverse health events among dual-eligible enrollees, 2016-2019 eReferences

Supplement 2. Data Sharing Statement
