# Supplementary material for: Heat Waves and Adverse Health Events Among Dually Eligible Individuals 65 Years and Older
Source: JAMA Health Forum. 2024 Nov 8;5(11):e243884. doi: 10.1001/jamahealthforum.2024.3884 (PMC11549656; doi:10.1001/jamahealthforum.2024.3884)
Supplement: Supplement 1. — eFigure 1. Local variation in heatwave days across ZCTAs within county boundaries in California eTable 1. Data sources utilized in this study eAppendix 1. Guide to R code eTable 2. Study sample selection eAppendix 2. Identification of outcomes eAppendix 3. Model specification eFigure 2. Nine climate regions by national centers of environmental information eAppendix 4. Identification of subgroups eTable 3. Type of service codes and place of service codes used to identify long-term service and supports (LTSS) use eAppendix 5. Subgroup sample selection eTable 4. Sample selection for subgroup analyses eTable 5. Zip code tabulation area (ZCTA)-level descriptive statistics, 2016-2019 eFigure 3. Association between lagged heatwaves and adverse health events among dual-eligible enrollees, 2016-2019 eReferences [file jamahealthforum-e243884-s001.pdf]

Supplemental Online Content

Kim H, Yoo EH, Senders A, et al. Heat waves and adverse health events among dually eligible individuals 65 years and older. *JAMA Health Forum*. 2024;5(11):e243884. doi:10.1001/jamahealthforum.2024.3884

eFigure 1. Local variation in heatwave days across ZCTAs within county boundaries in California

eTable 1. Data sources utilized in this study

eAppendix 1. Guide to R code

eTable 2. Study sample selection

eAppendix 2. Identification of outcomes

eAppendix 3. Model specification

eFigure 2. Nine climate regions by national centers of environmental information

eAppendix 4. Identification of subgroups

eTable 3. Type of service codes and place of service codes used to identify long-term service and supports (LTSS) use

eAppendix 5. Subgroup sample selection

eTable 4. Sample selection for subgroup analyses

eTable 5. Zip code tabulation area (ZCTA)-level descriptive statistics, 2016-2019

eFigure 3. Association between lagged heatwaves and adverse health events among dual-eligible enrollees, 2016-2019

eReferences

This supplemental material has been provided by the authors to give readers additional information about their work.

eFigure 1. Local variation in heatwave days across ZCTAs within county boundaries in California

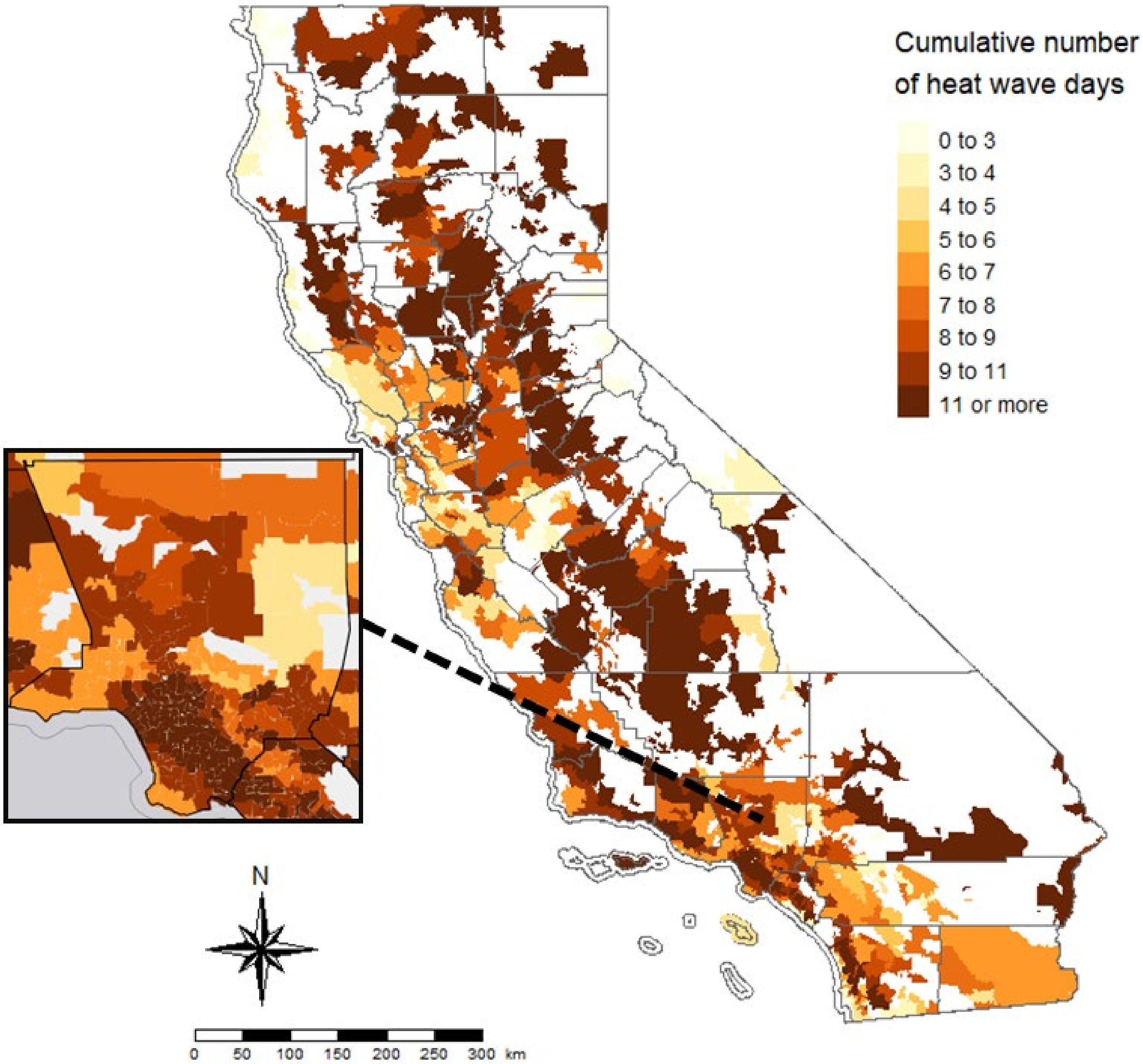

© 2024 Kim H et al. *JAMA Health Forum.*

eTable 1. Data sources utilized in this study

Source/Plan	Files	Identification of:	URL
Medicare FFS and MA (2016-2019)	Master Beneficiary Summary File (Base)	Enrollee age, sex, race, zip code, dual eligibility status, date of death	https://resdac.org/cms-data/files/mbsf-base
Medicare FFS (2016-2019)	Master Beneficiary Summary Files (27 and 30 CCW Chronic Conditions Segment)	Alzheimer's and related dementias	https://resdac.org/cms-data/files/mbsf-27-cc<br>https://resdac.org/cms-data/files/mbsf-30-cc
Medicare FFS (2016-2019)	Master Beneficiary Summary Files (Other Chronic Conditions Segment)	Substance use disorder (Alcohol use disorder, Opioid use disorder, Drug use disorders)	https://resdac.org/cms-data/files/mbsf-other-conditions
Medicare FFS (2016-2019)	Inpatient, Outpatient, Carrier, Skilled Nursing Facility, and Home Health Agency Fee-for-Service files	Mental health diagnoses	https://resdac.org/cms-data?tid_1%5B1%5D=1&tid%5B4931%5D=4931
Medicare MA (2016-2019)	Inpatient, Outpatient, Carrier, Skilled Nursing Facility, and Home Health Agency Encounter files	Dementia, substance use disorder, and mental health diagnoses	https://resdac.org/cms-data?tid_1%5B1%5D=1&tid%5B6056%5D=6056
Medicare FFS (2016-2019)	MedPAR	Hospitalizations and emergency department visits	https://resdac.org/cms-data/files/medpar
Medicare MA (2016-2019)	Inpatient (Encounter)	Hospitalizations and emergency department visits	https://resdac.org/cms-data/files/ip-encounter
Medicare FFS (2016-2019)	Outpatient (Fee-for-Service)	Emergency department visits	https://resdac.org/cms-data/files/op-ffs
Medicare MA (2016-2019)	Outpatient (Encounter)	Emergency department visits	https://resdac.org/cms-data/files/op-encounter
Medicaid T-MSIS Analytic Files (2016-2019)	Demographic and Eligibility Base File	Dual-eligibility, zip code	https://resdac.org/cms-data/files/taf-de
	Other Services File	HCBS utilization, substance use disorder, and mental health diagnoses	https://resdac.org/cms-data/files/taf-ot
	Long Term Care File	Nursing facility utilization	https://resdac.org/cms-data/files/taf-lt

© 2024 Kim H et al. *JAMA Health Forum.*

	Inpatient File	Nursing facility utilization, substance use disorder, and mental health diagnoses	https://resdac.org/cms-data/files/taf-lt
Oak Ridge National Laboratory Distributed Active Archive	Daymet: Daily Surface Weather Data on a 1-km grid for North America, Version 4 R1	Vapor pressure, maximum and minimum daily temp	https://daac.ornl.gov/about/
Robert Graham Center (2016)	Social Deprivation Index at the ZCTA level	Social deprivation index score	https://www.graham-center.org/maps-data-tools/social-deprivation-index.html

© 2024 Kim H et al. *JAMA Health Forum.*

eAppendix 1. Guide to R code

Overview

Sample R code to calculate average daily weather variables by ZCTA and to flag heatwave days is included in the supplemental material for this manuscript. The code was developed for parallel processing but could be adapted if parallel processing is not available.

Presumed Directory Structure

Our code presumes that daily weather files are stored in a single directory, and that US state and ZCTA shape files are each saved in separate directories. Shape files are available from www.census.gov.

Final Data structure

Together, the following scripts produce a single table that includes one row per ZCTA-year. The table contains columns for daily average maximum temperature, daily average minimum temperature, daily average relative humidity, ZCTA-level 90th and 97th percentile maximum temperature and minimum temperature thresholds, and a daily heatwave indicator.

R scripts

1_climate_data_by_zcta.R

This script calculates average daily weather variables by ZCTA for the continental US during warm months (May 1st through September 30th). To reduce processing time, this code processes one weather variable (e.g., max temperature) for one state during one year at a time. A separate table for each weather variable/state/year is saved into a single directory and bound in the next script.

2_bind_climate_vars_years.R

This script binds the yearly tables saved by the previous script into a single table for each weather variable by state. Care should be taken to make sure the list of states is complete and does not include any states that were not processed in the first script.

3_calculate_rh_flag_heat_waves.R

This script merges the weather variable tables saved by the previous step into a single table for each state. It then calculates daily average maximum and minimum temperatures and relative humidity. We used the esat() function from the plantecophys package to calculate relative humidity. The code then calculates the 90th and 97th percentile maximum and minimum temperatures for each ZCTA using all days (May 1st – September 30th) across all years. Finally, this script creates a heatwave flag. It uses a function that is stored in a separate R script to flag all ZCTA-days that meet the definition of a heatwave day.

© 2024 Kim H et al. *JAMA Health Forum.*

FlagHeatWave_Function.R

This script creates a function to flag heatwaves. It takes any of the heatwave threshold temperature columns as arguments, and then uses them to create heatwave flags for maximum or minimum temperatures. The function flags each day as being part of a heatwave (flag = 1) or not part of a heatwave (flag = 0)

The function takes four arguments:

- table - The table to flag heatwaves in. In our heatwave scripts, the table is called *state_temps*.
- days - Can be 2, 3, or 4 to flag heatwaves that are 2, 3, or 4 consecutive days above the threshold temperature.
- max_min – “tmax” if creating indicators for daily maximum temperature heatwaves, “tmin” if creating indicators for daily minimum temperature indicators.
- threshold - The column name where the threshold temps are located.
- flag_name - The name you want for the column that will store the heatwave flags. The function will append the day of the year to the end of the flag name.

Example: To create a heatwave flag in a column called "hw_flag_max_97_3_day_xxx” for heatwaves that are 3 days long, using the 97th percentile maximum temperature (stored in a column called "hw_max_97"), in a table called "temps", the function call would be written:

new_table <- hw_flag(table = temps, days = 3, max_min = “tmax”, threshold = "hw_max_99", flag_name = "hw_flag_max_99_3_day_")

4_bind_states

This script binds the state-level tables of weather variables and heatwave indicators to produce a single table that includes data for all states. This is the final script in the weather variable and heatwave indicator pipeline.

© 2024 Kim H et al. *JAMA Health Forum.*

eTable 2. Study sample selection

Sample selection step	Enrollees (N)	Change (N)	Change (%)
Age 65+, fully enrolled and dually eligible May-Sept	6,279,148		
Exclude enrollees living in US territories	6,273,667	-5,481	0.1%
Restrict to enrollees living in the same county May-Sept	6,233,705	-39,962	0.6%
Restrict to matching Medicaid ZCTA/dual-eligibility status	5,508,570	-725,135	11.6%
Restrict to ZCTAs with ≥ 1 enrollee during 2016-2019	5,448,499	-60,071	1.1%

© 2024 Kim H et al. *JAMA Health Forum.*

eAppendix 2. Identification of outcomes

Number of all-cause emergency department (ED) visits

Records for ED visits that result in an inpatient admission are found in the MedPAR and Medicare Inpatient Encounter files, otherwise ED visit claims are located in the Outpatient Fee-for-Service and Outpatient Encounter files. We identified ED visits with revenue center code (REV_CNTR) values of 0450-0459 (emergency room) or 0981 (professional fees-emergency room).

Number of all-cause hospitalizations

We used 2016 HEDIS specifications to identify hospitalizations (Inpatient Utilization—General Hospital/Acute Care).[1] Because MedPAR files lack claim-line variables (e.g. revenue center codes, facility type, etc.), we modified HEDIS specifications to identify acute fee-for-service hospitalization claims with the following criteria:

- Inpatient stay (CLM_TYPE_CD of 60 or 61) with a short stay provider indicator code (SSLSSNF = S)
- Inpatient stay (CLM_TYPE_CD of 60 or 61) in a critical access hospital (3rd and 4th digit of PRVDR_NUM = 13)

Heat-related ED visits and hospitalizations

We further classified ED visits and hospitalizations as heat-related if any of the following diagnosis codes were found in any position on a claim for the encounter: volume depletion (E86), electrolyte imbalance (E87), effects of heat and light (T67), exposure to excessive heat (X30), or exposure to sunlight (X32). [2,3]

Heat-specific ED visits and hospitalizations

We classified ED visits and hospitalizations as heat-specific if any of the following diagnosis codes were found in any position on a claim for the encounter: effects of heat and light (T67), exposure to excessive heat (X30), or exposure to sunlight (X32). [2]

New nursing facility placement

We defined nursing facility placements as any nursing facility use (as defined above) among enrollees who did not have any nursing facility use in the previous calendar year.

Deaths

We identified deaths using the enrollee DEATH_DT in the Medicare Master Beneficiary Summary File.

© 2024 Kim H et al. *JAMA Health Forum.*

eAppendix 3. Model specification

For all outcomes, we used the following Poisson model specification:

$$\log\left(\frac{Y_{id}}{n_{id}}\right) = \mu + \beta Heatwave_{id} + \theta X_{id} + \alpha_i$$

where Y_{id} is the outcome for ZCTA *i* on day *d* (taking place May-September, 2016-2019), offset for the size of the ZCTA's population on that day n_{id}. $Heatwave_{id}$ is a vector of indicators for whether the day *d* was a heatwave day or not, and β is the corresponding vector of coefficients. X_{id} is a vector of time-varying independent variables (daily relative humidity; annual proportion of ZCTA that is female; proportion of ZCTA that is aged 65-74, 75-84, or 85+; and indicators for day of the week (Sunday-Saturday), week-year of the study period, and federal holidays), and θ is the corresponding vector of coefficients. Finally, α_i are the ZCTA fixed effects. We clustered standard errors at the ZCTA level and calculated confidence intervals using the delta method.[4]

© 2024 Kim H et al. *JAMA Health Forum.*

eFigure 2. Nine climate regions by national centers of environmental information

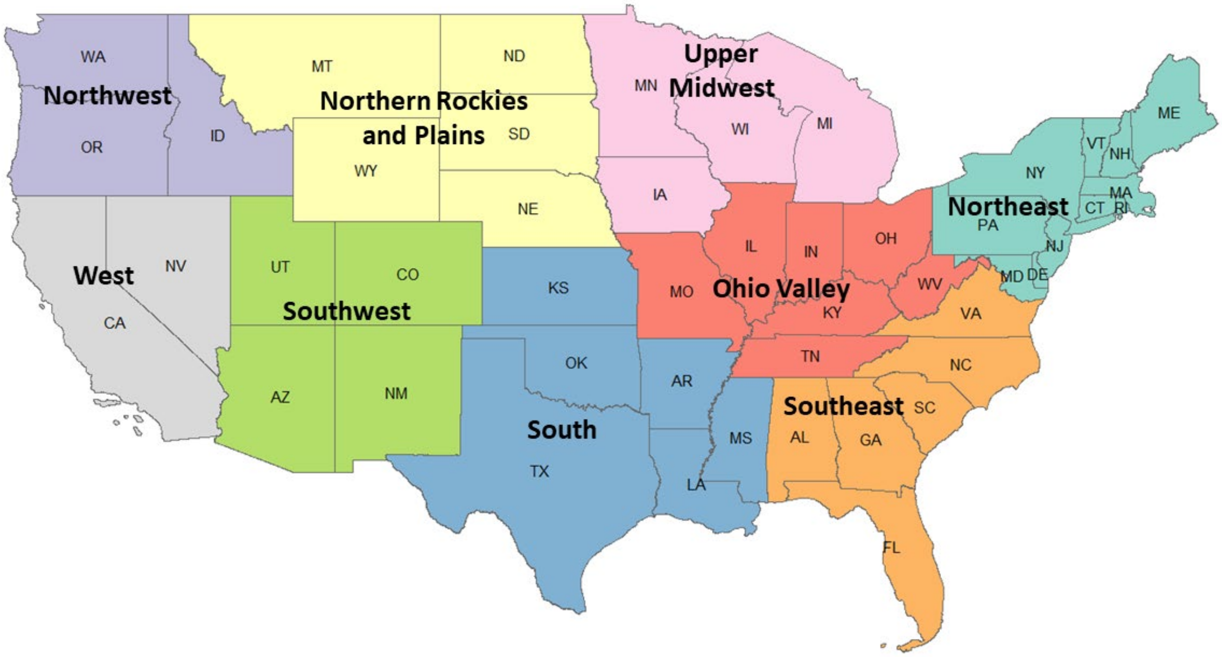

© 2024 Kim H et al. *JAMA Health Forum.*

eAppendix 4. Identification of subgroups

Alzheimer's disease and related dementias (ADRD)

We created a binary variable for whether an enrollee had claims for ADRD prior to the start of each study year (yes/no). For enrollees with Medicare fee-for-service in 2016 or 2017, we used chronic condition flags published by the Chronic Conditions Warehouse (CCW 27) to identify the presence of ADRD at the end of 2015 and 2016, respectively (yes/no). For enrollees in 2018 or 2019, we used the revised CCW 30 flags to identify the presence of ADRD at the end of 2017 and 2018, respectively.

CCW flags are not available for Medicare Advantage. For enrollees in this program we used the CCW 30 algorithm to identify ADRD using Medicare Advantage claims. Because the algorithm requires a two-year lookback, we could only include Medicare Advantage enrollees in 2018 or 2019 in the ADRD sub-analysis.

Because a high proportion of individuals with ADRD reside in nursing facilities with interior temperature control, we restricted this analysis to community dwelling individuals with and without ADRD. To accurately identify community dwelling individuals, we also excluded individuals who lived in five states with potentially unreliable nursing facility claims data per the DQ Atlas (AR, ID, MS, NH, and RI).[5]

Mental health diagnosis

We created a categorical variable for mental health status (none, mild-to-moderate, or severe mental illness). To identify enrollees in Medicare fee-for-service and Medicare Advantage with a mental health condition prior to each study year, we first used the Agency for Healthcare Research and Quality's Clinical Classifications Software Refined (CCSR) to identify a broad list of mental health diagnostic codes.[6] We began with all ICD-10 codes included in the CCSR body system "Mental, Behavioral, and Neurodevelopmental Disorders". We then excluded codes related to a physiologic condition, substance use, or childhood condition. Our final set of ICD-10 codes included F20-69 and F90-99 (including subcategory codes and excluding F55, F64.2, F93, F94.8, F94.9, and F98), as well as all the codes in the CCSR Category MBD012 "Suicidal ideation/attempt/intentional self-harm".

We defined enrollees with severe mental illness (SMI) as those with at least 1 inpatient claim OR 2 other non-drug claims of any service type over a two-year period with any diagnosis of schizophrenia (F20, F25), bipolar I (F30, F31.0-F31.78), or major depressive disorder (F32.2, F32.3, F33.2, F33.3).

© 2024 Kim H et al. *JAMA Health Forum.*

We defined enrollees with a mild-to-moderate mental health diagnosis as those with at least 1 inpatient claim OR 2 other non-drug claims of any service type over a two-year period with any mental health diagnosis code that did not fall under our definition of SMI.

Enrollees who did not meet the criteria for SMI or a mild-to-moderate diagnosis were categorized as "none". Because our algorithms require a two-year lookback, we could only include enrollees in 2018 or 2019 in the mental health sub-analysis.

Race and ethnicity

There are six categories of race and ethnicity available in Medicare data: American Indian/Alaska Native, Asian/Pacific Islander, Black, Hispanic, non-Hispanic white, and Other. An enrollee's race/ethnicity is identified from information used by the Social Security Administration and an algorithm developed by the Research Triangle Institute (RTI), which determines whether enrollee names are likely Asian or Hispanic in origin.[7] The RTI race code has high sensitivity and specificity compared to self-reported race and ethnicity.[8] When we restricted to ZCTAs with at least 1 enrollee in each racial/ethnic subgroup in each year 2016-2019, small sample sizes limited our final subgroup analysis to individuals who were identified as Asian/Pacific Islander, Black, Hispanic, and non-Hispanic white.

Region

We categorized ZCTAs into nine climatically consistent US regions: Northeast, Northern Rockies and Plains, Northwest, Ohio Valley, South, Southeast, Southwest, Upper Midwest, West.[9,10]

Rurality

We used 2010 Rural-Urban Commuting Area (RUCA) codes to categorize ZCTAs as urban (1.0, 1.1, 2.0, 2.1, 3.0, 4.1, 5.1, 7.1, 8.1, 10.1) or rural (4.0, 4.2, 5.0, 5.2, 6.0, 6.1, 7.0, 7.2, 7.3, 7.4, 8.0, 8.2, 8.3, 8.4, 9.0, 9.1, 9.2, 10.0, 10.2, 10.3, 10.4, 10.5, 10.6).[11]

Social deprivation index (SDI) score

We created a binary variable of social deprivation based on the 2016 ZCTA-level SDI score.[12] We assigned each ZCTA a SDI score and then categorized each ZCTA as either above (higher social deprivation) or below (lower social deprivation) the sample median.

Substance use disorder (SUD)

We created a binary variable for whether an enrollee had claims for SUD prior to the start of each study year (yes/no). Both Medicare and Medicaid cover behavioral health services for dually eligible individuals. Therefore, we used both Medicare and Medicaid claims to identify the presence of SUD in

© 2024 Kim H et al. *JAMA Health Forum.*

our analytic cohort. We used a combination of the CCW Other Chronic Condition algorithms for Alcohol Use Disorders, Drug Use Disorders, and Opioid Use Disorder #1 to identify the presence of SUD in Medicare fee-for-service and Medicare Advantage enrollees.[13] Because the algorithms require a two-year lookback, we could only include enrollees in 2018 or 2019 in the SUD sub-analysis.

Long-term Services Supports (LTSS)

We created a categorical variable for LTSS use (none, HCBS, or nursing facility resident) in a given study year.

To identify HCBS users, we followed the methodological guidance for *Identifying Home and Community-Based Services and the Enrollees Who Use Them in the TAF* published by CMS.[14] Step three in the five-step process instructs researchers to use type of service codes (TOS_CD) to identify HCBS claims but is not prescriptive about which codes should be employed. The TOS_CDs used to identify HCBS in this study are presented in eTable 3. Because this method of identifying HCBS use relies most heavily on procedure codes and type of service codes in the TAF Other Services File, we replicated DQ Atlas data quality assessments for these two variables for all dual eligible individuals from 2016-2019. In any given study year, all states had <= 10% missing procedure codes or type of service codes. We therefore did not exclude any states from the LTSS subgroup analysis based on these criteria.

We used two methods to identify enrollees who resided in a nursing facility during May-September of a given year. First, we used a combination of type of service code (TOS_CD) and service dates (SRVC_BGN_DT, SRVC_END_DT) on claims in the Long-Term Care Analytic Files to identify nursing facility claims. We used the following type of service codes to identify nursing facility stays: 009, 045-047, 059. We used the Claim Beginning Date of Service (SRVC_BGN_DT) and the Claim Ending Date of Service (SRVC_END_DT) to calculate the length of nursing facility stays. We chose not to use discharge dates on long-term care claims due to data quality issues documented by the Center for Medicaid and Chip Services.[5] For each enrollee we grouped claims with consecutive or overlapping dates of service into nursing facility encounters. We defined nursing facility residents as individuals with nursing facility encounters from at least May-September in a given year with no more than a 10-day gap.

Second, we followed methodology published by CMS that is used to assign a service category variable to TAF claims. Beginning with the 2021 data release, this federally assigned service category (FASC) variable is meant to provide users with an alternative method to identify and select records for services that tend to be reported inconsistently across states. We identified nursing facility claims by applying the algorithm outlined in CMS Methodology Brief #5241 Appendix A for FASC category 6 (Nursing Facility).[15] We then

grouped claims for each enrollee into encounters as described above. Enrollees who met our criteria for nursing facility resident under either approach were categorized as a nursing facility resident.

We replicated DQ Atlas data quality assessments for type of service codes in the TAF Long-term File for all dual eligible individuals from 2016-2019. In any given study year, all states had <= 10% missing type of service codes. For this sub-analysis we also excluded five states that DQ Atlas has flagged for having lower long-term claims volume than expected (AR, ID, MS, NH, RI).

© 2024 Kim H et al. *JAMA Health Forum.*

eTable 3. Type of service codes and place of service codes used to identify long-term service and supports (LTSS) use

Type of LTSS	Type of Service Codes	Place of Service Codes
Nursing Facility	009, 045-047, 059	Not applicable
HCBS	016, 017, 018, 019, 020, 021, 043, 051, 053, 062, 063, 064, 065, 066, 067, 068, 069, 070, 071, 072, 073, 074, 075, 076, 077, 078, 079, 080, 081, 082, 083, 115, 144	Any POS_CD
	054	04, 12, 13, 14, 16, 33, 34, 55, 56
	022	04, 12, 13, 14, 16, 33, 34, 55, 56

© 2024 Kim H et al. *JAMA Health Forum.*

eAppendix 5. Subgroup sample selection

We used the same study sample criteria from our main analysis to identify each of our subgroup study samples, with two exceptions. After restricting the sample to those with matching Medicare/Medicaid ZCTA and dual eligibility status, we excluded enrollees in years that we could not include for that particular analysis due to data limitations (see Subgroup Identification for ADRD, Mental health, and SUD). We then restricted to ZCTAs with at least 1 enrollee in each subgroup strata between 2016-2019 (eTable 4).

eTable 4. Sample selection for subgroup analyses

Analysis	Selection step	Enrollees (N)	Change (N)	Change (%)	Excluded states
Common selection steps for all analyses					
	Fully enrolled duals May-Sept	6,279,148			
	Exclude enrollees living in US territories	6,273,667	-5,481	0.1%	
	Restrict to same Medicare county May-Sept	6,233,705	-39,962	0.6%	
	Restrict to matching Medicaid ZCTA/dual status	5,508,570	-725,135	11.6%	
Additional selection steps based on sub-analysis					
ADRD	Exclude MA enrollees in 2016-2017	5,080,404	-428,166	7.8%	
	Exclude states with poor quality Medicaid data	4,966,494	-113,910	2.2%	AR, ID, MS, NH, RI
	Restrict to ≥ 1 enrollee in each ADRD strata	4,795,167	-171,327	3.5%	WY
SUD	Exclude FFS/MA enrollees in 2016-2017	4,294,887	-1,213,683	22.0%	RI
	Restrict to ≥ 1 enrollee in each SUD strata	4,157,884	-137,003	3.2%	
Region, rurality, social deprivation	Restrict to ≥ 1 enrollee per ZCTA	5,448,499	-60,071	1.1%	~none
LTSS	Exclude states with poor quality Medicaid data	5,378,714	-129,856	2.4%	AR, ID, MS, NH, RI
	Restrict to ≥ 1 enrollee in each LTSS strata	4,877,522	-501,192	9.3%	WY
Mental health	Exclude FFS/MA enrollees in 2016-2017	4,294,887	-1,213,683	22.0%	RI

© 2024 Kim H et al. *JAMA Health Forum.*

	Restrict to ≥ 1 enrollee in each MH strata	4,123,699	-171,188	4.0%	
Race/ethnicity	Restrict to ≥ 1 enrollee in each strata	3,805,510	-1,703,060	30.9%	AR, ID, NE, RI, WY
Exclude Incomplete MA Data	Exclude poor quality MA contracts	4,539,656	-968,914	17.5%	~none
	Restrict to ≥ 1 enrollee per ZCTA	4,480,132	-59,524	1.3%	

ADRD: Alzheimer's dementia and related disorders; FFS: Fee-for-service; MA: Medicare Advantage; LTSS: Long-term services and supports; SUD: Substance use disorder; ZCTA: Zip code tabulation area

© 2024 Kim H et al. *JAMA Health Forum.*

eTable 5. Zip code tabulation area (ZCTA)-level descriptive statistics, 2016-2019

	ZCTAs (States)	Population size[a]	Mean Proportion Across ZCTAs (SD)			
			Female	65-74 years	75-84 years	≥ 85 years
**Main analysis**	28,404 (51)	5,448,499	0.66 (0.07)	0.48 (0.10)	0.32 (0.06)	0.20 (0.08)
**Subgroup**						
Alzheimer's or Related Dementia[b]						
No	17,420 (45)	4,212,655	0.65 (0.06)	0.55 (0.09)	0.32 (0.06)	0.14 (0.06)
Yes	17,420 (45)	940,356	0.71 (0.11)	0.21 (0.11)	0.36 (0.11)	0.43 (0.14)
Substance Use Disorder[c]						
No	18,751 (50)	3,893,606	0.67 (0.06)	0.47 (0.09)	0.32 (0.05)	0.20 (0.08)
Yes	18,751 (50)	377,382	0.52 (0.16)	0.65 (0.17)	0.26 (0.13)	0.09 (0.10)
Rurality						
Rural	11,651 (49)	896,554	0.67 (0.09)	0.48 (0.11)	0.30 (0.08)	0.22 (0.10)
Urban	16,831 (51)	4,569,766	0.66 (0.06)	0.48 (0.10)	0.32 (0.05)	0.20 (0.08)
Social Deprivation Index[d]						
Higher Deprivation	5,047 (50)	2,705,939	0.64 (0.05)	0.51 (0.08)	0.32 (0.05)	0.17 (0.05)
Lower Deprivation	23,276 (51)	2,844,219	0.68 (0.07)	0.45 (0.11)	0.32 (0.07)	0.23 (0.09)
Long-term Services and Supports Use						
None	14,186 (45)	3,653,450	0.64 (0.07)	0.55 (0.10)	0.31 (0.06)	0.14 (0.07)
Home and Community-based Services	14,186 (45)	1,383,067	0.70 (0.08)	0.38 (0.14)	0.37 (0.08)	0.26 (0.10)
Nursing facility	14,186 (45)	647,239	0.71 (0.12)	0.24 (0.13)	0.32 (0.11)	0.45 (0.16)
Mental Health[e]						
None	17,352 (50)	2,649,760	0.61 (0.07)	0.49 (0.10)	0.32 (0.06)	0.19 (0.07)
Mild/Moderate	17,352 (50)	1,452,470	0.74 (0.07)	0.44 (0.12)	0.32 (0.07)	0.23 (0.10)
Severe	17,352 (50)	342,403	0.67 (0.14)	0.60 (0.17)	0.28 (0.13)	0.12 (0.11)
Race/ethnicity[f]						
Asian/Pacific Islander	6,673 (46)	571,062	0.60 (0.07)	0.43 (0.11)	0.38 (0.08)	0.20 (0.08)
Black	6,673 (46)	694,195	0.67 (0.09)	0.54 (0.11)	0.30 (0.07)	0.16 (0.07)
Hispanic	6,673 (46)	1,060,760	0.65 (0.07)	0.49 (0.09)	0.35 (0.06)	0.16 (0.06)
Non-Hispanic White	6,673 (46)	1,481,159	0.67 (0.07)	0.46 (0.12)	0.30 (0.06)	0.24 (0.10)
US Region[g]						
Northeast	5,968 (12)	1,381,295	0.66 (0.06)	0.46 (0.10)	0.32 (0.05)	0.22 (0.09)
Northern Rockies	886 (5)	41,953	0.69 (0.10)	0.45 (0.15)	0.28 (0.09)	0.27 (0.14)
Northwest	1,057 (4)	144,977	0.65 (0.07)	0.49 (0.10)	0.33 (0.07)	0.18 (0.08)
Ohio Valley	5,916 (7)	725,518	0.68 (0.07)	0.49 (0.10)	0.30 (0.06)	0.21 (0.09)
South	3,853 (6)	561,799	0.68 (0.07)	0.45 (0.09)	0.33 (0.06)	0.21 (0.07)
Southeast	4,268 (6)	865,887	0.69 (0.06)	0.46 (0.09)	0.33 (0.06)	0.21 (0.07)

© 2024 Kim H et al. *JAMA Health Forum.*

Southwest	1,297 (4)	209,888	0.63 (0.07)	0.56 (0.09)	0.29 (0.06)	0.15 (0.07)
Upper Midwest	3,294 (4)	339,002	0.68 (0.08)	0.51 (0.13)	0.28 (0.07)	0.21 (0.12)
West	1,865 (3)	1,184,172	0.61 (0.05)	0.50 (0.08)	0.33 (0.04)	0.17 (0.05)
Exclude Incomplete MA Data	27,912 (51)	4,480,132	0.66 (0.07)	0.47 (0.11)	0.32 (0.07)	0.21 (0.09)

[a] Individuals may belong to more than one strata over the course of the study

[b] Analytic years for this analysis included Medicare Fee-for-Service (FFS) 2016-2019 and Medicare Advantage 2018-2019.

[c] Analytic years for this analysis included Medicare FFS and Medicare Advantage 2018-2019.
[d] ZCTAs were categorized as higher deprivation (above) or lower deprivation (below) based on the Social Deprivation Index score national median.

[e] Analytic years for this analysis included Medicare FFS and Medicare Advantage 2018-2019.

[f] For a ZCTA to be included in the study it must have had at least 1 resident from each racial/ethnic category in all year study years; sample size constraints restricted the number of racial/ethnic categories we could include in our analysis.
[g] Northeast: CT, DC, DE, MA, MD, ME, NH, NJ, NY, PA, VT; Northern Rockies: MT, ND, SD; Northwest: AK, OR, WA; Ohio Valley: IL, IN, KY, MO, OH, TN, WV; South: AR, KS, LA, MS, OK, TX; Southeast: AL, FL, GA, NC, SC, VA; Southwest: AZ, CO, NM, UT; Upper Midwest: IA, MI, MN, WI; West: CA, HI, NV

© 2024 Kim H et al. *JAMA Health Forum.*

eFigure 3. Association between lagged heatwaves and adverse health events among dual-eligible enrollees, 2016-2019

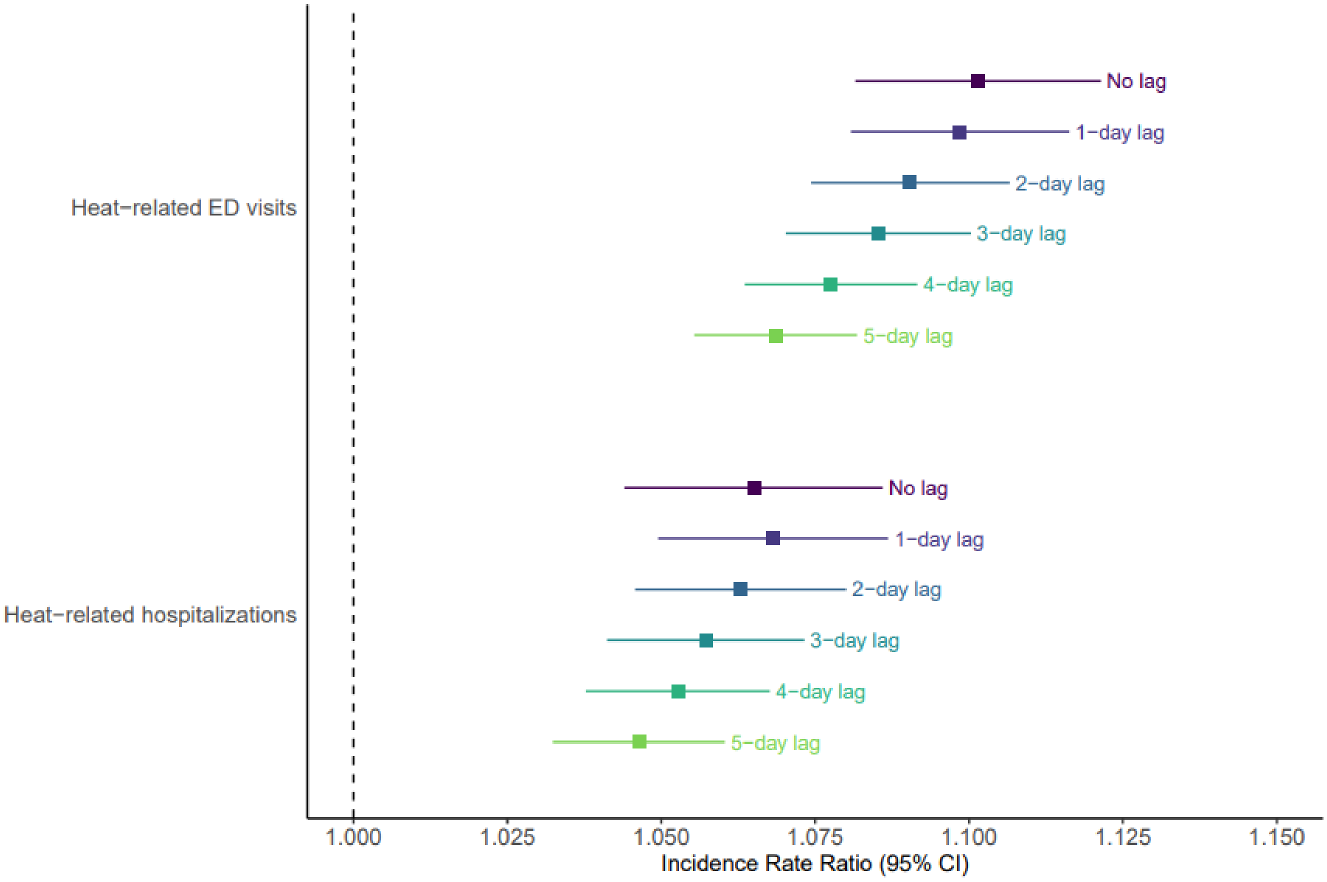

© 2024 Kim H et al. *JAMA Health Forum.*

eReferences

1. National Committee for Quality Assurance. HEDIS 2016 Volume 2 (epub). Accessed July 13, 2023. https://store.ncqa.org/hedis-2016-volume-2-epub.html

2. Owens P, Barrett M, McDermott K. Emergency Department Visits for Diagnoses Directly Indicating Heat Exposure: Variation Across Counties in the United States, 2016–2020. *Agency for Healthcare Research and Quality Healthcare Cost and Utilization Project*. Published online December 8, 2022. https://hcup-us.ahrq.gov/reports/ataglance/HCUPanalysisHeatExposureEDvsts.pdf.

3. Nori-Sarma A, Milando C, Weinberger KR, Hess JJ, Errett NA, Wellenius GA. Association Between the 2021 Heat Wave in Portland, Oregon, and Seattle, Washington, and Emergency Department Visits. *JAMA*. 2022;328(23):2360-2362. doi:10.1001/jama.2022.20665

4. Rosner B. *Fundamentals of Biostatistics*. 8th ed. Cengage Learning; 2015. Accessed April 24, 2024. https://www.cengage.com/c/fundamentals-of-biostatistics-8e-rosner/9781305268920/

5. Center for Medicare & Medicaid Services. DQ Atlas. Accessed February 2, 2022. https://www.medicaid.gov/dq-atlas/welcome

6. Clinical Classifications Software Refined (CCSR) for ICD-10-CM Diagnoses. Accessed April 1, 2022. https://www.hcup-us.ahrq.gov/toolssoftware/ccsr/dxccsr.jsp

7. ResDAC. Research Triangle Institute (RTI) Race Code. 2023. https://resdac.org/cms-data/variables/research-triangle-institute-rti-race-code

8. Jarrín OF, Nyandege AN, Grafova IB, Dong X, Lin H. Validity of Race and Ethnicity Codes in Medicare Administrative Data Compared With Gold-standard Self-reported Race Collected During Routine Home Health Care Visits. *Medical Care*. 2020;58(1):e1-e8. doi:10.1097/MLR.0000000000001216

9. U.S. Climate Regions | Geographical Reference Maps | National Centers for Environmental Information (NCEI). Accessed April 10, 2024. https://www.ncei.noaa.gov/access/monitoring/reference-maps/us-climate-regions

10. Karl T, Koss WJ. *Regional and National Monthly, Seasonal, and Annual Temperature Weighted by Area, 1895-1983*. National Climatic Data Center (U.S.); 1984.

11. Patterson D, Andrilla H. WWAMI Rural Health Research Center: Rural Urban Commuting Area Codes Data. Accessed April 10, 2024. https://depts.washington.edu/uwruca/ruca-uses.php

12. Social Deprivation Index (SDI). Accessed September 27, 2021. https://www.graham-center.org/rgc/maps-data-tools/sdi/social-deprivation-index.html

13. Other Chronic Health, Mental Health, and Potentially Disabling Conditions. Chronic Conditions Data Warehouse. Accessed April 10, 2024. https://www2.ccwdata.org/condition-categories-other

14. Greener E, Carpenter A, Nolan L. *Identifying Home and Community-Based Services and the Enrollees Who Use Them in the TAF*. CMS; 2023.

© 2024 Kim H et al. *JAMA Health Forum.*

15. Hula L, Parker J, Proctor K, Galecki J, Fokar A. *Assigning TAF Records to a Federally Assigned Service Category*. CMS; 2021. https://www.medicaid.gov/dq-atlas/landing/resources/downloads

© 2024 Kim H et al. *JAMA Health Forum.*
