# Supplementary material for: Heat Waves and Adverse Health Events Among Dually Eligible Individuals 65 Years and Older
Source: JAMA Health Forum. 2024 Nov 8;5(11):e243884. doi: 10.1001/jamahealthforum.2024.3884 (PMC11549656; doi:10.1001/jamahealthforum.2024.3884)
Supplement: Supplement 2. — Data Sharing Statement [file jamahealthforum-e243884-s002.pdf]

# Data Sharing Statement

Kim. Heat Waves and Adverse Health Events Among Dually Eligible Individuals 65 Years and Older. *JAMA Health Forum*. Published November 08, 2024.
doi:10.1001/jamahealthforum.2024.3884

**Data**
**Data available:** No

**Additional Information**
**Explanation for why data not available:** Our data sources are not allowed to be shared per CMS rule
